# Reproducibility of meta-analytic results in systematic reviews of interventions: meta-research study

**DOI:** 10.1136/bmjmed-2025-002024

**Published:** 2025-11-30

**Authors:** Phi-Yen Nguyen, Joanne E McKenzie, Zainab Alqaidoom, Daniel G Hamilton, David Moher, Matthew J Page

**Affiliations:** 1 Methods in Evidence Synthesis Unit, School of Public Health and Preventive Medicine, Monash University, Melbourne, VIC, Australia; 2 Centre for Journalology, Methodological and Implementation Research Program, Ottawa Hospital Research Institute, Ottawa, ON, Canada; 3 School of Epidemiology and Public Health, University of Ottawa Faculty of Medicine, Ottawa, ON, Canada

**Keywords:** Public health, Systematic Review

## Abstract

**Objective:**

To determine how often meta-analyses of effects of interventions are reproducible.

**Design:**

Meta-research study.

**Setting:**

Systematic reviews with meta-analyses of the effects of health, social, behavioural, or educational interventions indexed in five databases (PubMed, Science Citation Index, Social Sciences Citation Index, Scopus, and Education Collection), 2 November to 2 December 2020.

**Population:**

296 reviews meeting the inclusion criteria formed the overall sample of the study. 175/296 (59%) reviews included a forest plot from Review Manager and were considered inherently reproducible. The remaining 121/296 (41%) reviews constituted the reproduction sample.

**Main outcome measures:**

Original review authors were contacted to obtain meta-analysis data files, and analytic code used to generate the first reported (index) meta-analysis; if not provided, the necessary data and statistical details of the meta-analysis methods were extracted from the review. Two investigators independently reproduced each review’s first reported meta-analysis using the original computational steps and analytic code. Meta-analyses were classified as fully reproducible if the difference between the original and reproduced summary estimates and 95% confidence interval (CI) widths was less than 10%. Differences in meta-analysis results were classified as meaningful if there was a change in direction of the summary effect estimate or if the 95% CI included the null, which may alter the interpretation of the results.

**Results:**

22 authors provided data files or analytic code, or both. 104 meta-analyses (86%) were fully reproducible, seven (6%) were not fully reproducible, and 10 (8%) had insufficient data available to attempt reproduction. No meaningful differences were found in the reproduced meta-analytic results that might alter their interpretation (eg, changes in the direction of summary effect estimate or if the 95% CI included the null).

**Conclusions:**

The findings of the study suggested that the results of meta-analyses could be reliably replicated if the original data or analytic code, or both, could be obtained, or if the necessary data were accessible in the review. Few systematic reviewers responded to requests to share data or code. Making data files and analytic code publicly available will facilitate future investigations of reproducibility.

**WHAT IS ALREADY KNOWN ON THIS TOPIC**

- The results of meta-analyses should be reproducible (ie, others should obtain sufficiently similar results when reanalysing the same data with the same methods).
- No study has evaluated the reproducibility of meta-analyses of the effects of interventions, the most common type of meta-analysis in health research.

**WHAT THIS STUDY ADDS**

- Results of meta-analyses might be reliably replicated if the original data or analytic code, or both, could be obtained, or if the necessary data were accessible in the review.
- The response rate from the original systematic reviewers for sharing of data or analytic code was low
- Recommendations to systematic reviewers and other stakeholders to make this process more efficient are provided.

**HOW THIS STUDY MIGHT AFFECT RESEARCH, PRACTICE, OR POLICY**

- Making data files and analytic code publicly available will facilitate future investigations of reproducibility of meta-analyses and allow updates of reviews to be completed more quickly.
- Greater initiative and transparency in sharing of data and analytic code when reporting meta-analyses are needed.
- Future research should focus on whether reproducibility, or lack of reproducibility, is influenced by other processes in a systematic review, such as literature search or eligibility screening.

## Introduction

 Systematic reviews are a type of review where authors try to systematically identify and synthesise all relevant evidence on a research topic.^1^ A meta-analysis is a statistical technique commonly used in systematic reviews to combine estimates of effect from multiple studies into one summary effect estimate.^2^ In systematic reviews of interventions, a meta-analysis provides a quantitative summary of the benefits and harms of an intervention, how consistent the effects are across studies, and what factors might modify the magnitude of the effects, which can inform clinical practice guidelines and health policies. Given their potential to influence patient care, reproducibility of the results of meta-analyses is important.

Although the definition of reproducibility varies,^3^ the general consensus is that achieving reproducibility refers to obtaining the same, or reasonably similar, results when reanalysing data collected for a previous study, based on the same computational steps and analytic code as the original study.^4 5^ Results of an attempt to reproduce a meta-analysis can help users judge the validity and reliability of the original meta-analytic results before using them to guide clinical or policy decisions.^6^

Reproducing meta-analyses can be challenging. Reports of meta-analyses often lack details about the statistical methods^7^,^10^ and therefore the unique meta-analysis method used might not be identifiable. For example, if an author reports that they used a random effects meta-analysis without further details, numerous between-study variance estimators and interval methods for the summary effect could have been used.^11 12^ In some cases, the summary statistics used to calculate individual study effect estimates are not reported.^7 13 14^ In these scenarios, assumptions might need to be made about the data and meta-analysis methods in attempts to reproduce the results. If the assumed methods are different from the original data and methods, the reproduced and original meta-analytic results may differ.^15^,^18^

Several studies have evaluated the reproducibility of meta-analyses of effects in psychology experiments^19^,^21^ and of diagnostic test accuracy studies.^22^ To our knowledge, no study has evaluated the reproducibility of meta-analyses of the effects of interventions, which are the most common type of meta-analysis of health research.^23^ This study aims to look at this research gap by using a sample of systematic reviews of the effects of interventions.

### Objectives

The primary objective was to examine the extent of variation in results when we independently reproduced meta-analyses in our sample with the same data, computational steps, and analytic code (if available) as reported in the original review. Secondary objectives were to: determine how often meta-analyses were inherently reproducible based on the software used and publicly available data; examine the availability and ease of request for data and code for reproducing meta-analyses; and record common challenges encountered when reproducing meta-analyses.

## Methods

This study was conducted as one of a group of studies in the REPRISE (Reproducibility and Replicability In Syntheses of Evidence) project. The REPRISE project investigates various aspects of the transparency, reproducibility, and replicability of systematic reviews with meta-analysis of the effects of health, social, behavioural, and educational interventions.^7 24 25^ The methods for all of the studies were prespecified in the same protocol.^5^ Online supplemental file S1 outlines deviations from the study protocol.

### Eligibility criteria, search, and selection of systematic reviews

Systematic reviews included in a previous study assessing completeness of reporting in systematic reviews formed the initial sample for our study.^7^ These reviews were identified from a search of PubMed, Science Citation Index, and Social Sciences Citation Index through the Web of Science service, Scopus through Elsevier, and the Education Collection through ProQuest for systematic reviews with meta-analyses of the effects of a health, social, behavioural, or educational intervention, indexed between 2 November and 2 December 2020. Two authors independently screened the titles and abstracts of the first 2000 randomly selected records, and evaluated their full texts against the eligibility criteria until the target sample size of 300 systematic reviews was reached (see Nguyen et al^7^ for further details). We then excluded reviews that had been retracted since we had identified them and those where a bayesian meta-analysis was used. We restricted our study to meta-analyses undertaken in the frequentist framework because this framework is most commonly used. This selection formed the “overall sample” for our study.

### Selection of reviews for reproduction

We considered all reviews that used Review Manager (RevMan, a systematic review production software from the Cochrane Collaboration) to conduct meta-analyses, and which included a forest plot generated from RevMan, as inherently reproducible. Forest plots generated by RevMan almost always display the study level summary statistics (eg, mean and standard deviation) and the statistical methods used. Therefore, a reproducibility attempt for such meta-analyses would always return the same results as presented in the original review.

Reviews that did not include a forest plot generated from RevMan comprised the “reproduction sample.” In each review in this sample, we attempted to reproduce the first meta-analysis that was mentioned in the abstract or the results section (ie, index meta-analysis).

### Correspondence with original systematic reviewers

For each systematic review included in the reproduction sample, we invited the corresponding author of the review to share the data file or files and analytic code so that we could attempt to reproduce the meta-analysis. We sent emails to the corresponding authors of the reviews on 2 November 2021. Up to three reminders were sent two weeks apart if no response was received. The authors were provided with a document explaining the process of our reproducibility study (online supplemental data S2), along with a request to provide the summary statistics underlying the index meta-analysis in a usable format (eg, CSV file) and the analytic code. The corresponding authors of all reviews included in this reproducibility study gave informed consent before participation. All data provided by participants were de-identified and stored on a secure cloud based storage server (Google Drive).

### Data extraction

For each index meta-analysis that we planned to reproduce, we extracted the aggregate study level data for the index meta-analysis from relevant tables and figures (eg, forest plot) in the review if the corresponding author did not respond to our request for their data or had not already made their data files publicly available. Aggregate study level data included individual study summary statistics (ie, for continuous data, means, standard deviations, and sample sizes, and for binary outcomes, number of events and sample sizes) and study effect estimates (eg, standardised mean difference) and measures of precision (eg, 95% confidence interval (CI) or standard error). We also recorded the software and its version number, the package (for R) or command (for Stata) used for the meta-analysis, and details of the statistical methods, such as meta-analysis model (eg, random effects or fixed effect model), meta-analysis method (eg, inverse variance or Mantel-Haenszel method), heterogeneity variance estimator used, if applicable (eg, DerSimonian-Laird or restricted maximum likelihood estimator), and the method of continuity correction if the summary statistics contained zero cells. We recorded any information about the analysis methods, regardless of where it appeared in the review (eg, the methods section, table footnotes, or forest plots).

Data extraction was carried out by one investigator (P-YN) with a standardised data extraction form in Microsoft Excel. We did not verify the data we extracted from the systematic reviews against data reported in the primary studies.

### Reproduction of meta-analyses

We attempted to reproduce the index meta-analyses of all reviews in the reproduction sample by using the data, analytical code, and statistical methods of the original review. If analytic code used to generate the meta-analytic results was available, we used the code without modification. For studies where code was not available or did not work, we attempted to recreate the code based on a template that we created for each software, customised to the statistical details specified in the article (online supplemental data S3).

For index meta-analyses where we obtained the necessary data, two investigators (P-YN and ZA or MJP) independently carried out a reanalysis of the meta-analysis. If a discrepancy was found between the results of the investigators' analyses, the investigators discussed potential reasons for the discrepancies and repeated the meta-analysis with necessary adjustments until sufficiently similar results were obtained. We followed an algorithm to select the appropriate software, software packages, and statistical methods when these details were not available (online supplemental data S4).

If summary statistics were available for each study included in the meta-analysis, we attempted to reproduce the meta-analysis with these values. If only effect estimates and measures of precision were available, we attempted to reproduce the meta-analysis with these values, with the inverse variance method (online supplemental data S4). For meta-analyses of ratio measures (eg, odds ratios, risk ratios, or hazard ratios), we log transformed the effect estimates and 95% CIs before analysis.

### Comparison of results from original and reproduced meta-analyses

The results of interest for comparison of the original and reproduced meta-analyses were the meta-analysis summary estimate, its CI, and the P value from the statistical test of the summary estimate. We defined reproducibility based on these criteria: results fully reproducible (ie, <10% difference was seen between the original and reproduced summary effect estimate and the width of the 95% CI reported in the original review); results not fully reproducible (ie, a difference ≥10% observed between the original and reproduced meta-analytic effect estimate or the width of the 95% CI reported in the original review); or results could not be reproduced because of missing information.

We conducted a sensitivity analysis with a threshold of 5% instead of 10% difference. We defined a meaningful difference between the original and reproduced results as when the original and reproduced summary estimates had the opposite direction of effect (ie, above *v* below the null effect; one for ratio measures and zero for difference measures) or the original 95% CI included the null whereas the reproduced 95% CI did not, or vice versa. Two investigators (P-YN and MJP) independently evaluated whether the observed differences were meaningful based on these definitions. Discrepancies in the evaluations were resolved by discussion.

#### Reproducibility of results

We calculated these statistics with exact binomial 95% CIs^26^: frequency and percentage of systematic reviews where summary statistics or effect estimates, analytic code, and statistical details for the index meta-analysis were available; frequency and percentage of index meta-analyses within each of the reproducibility categories, grouped by whether the original reviewer was involved (ie, provided the data file or analytic code, or both); and frequency and percentage of index meta-analyses with a meaningful difference between the original and reproduced results.

#### Comparison of meta-analysis estimates and confidence intervals

We constructed Bland-Altman plots to assess the agreement between the reproduced and original summary effect estimates. Differences between the reproduced and original summary effect estimates were plotted against their mean, together with the mean of the differences and the 95% limits of agreement.^27^ Plots were generated separately for meta-analyses of standardised mean differences, log risk ratios (log(RR)), log odds ratios (log(OR)), and log hazard ratios (log(HR)). In each Bland-Altman plot, we determined whether reproduction was achievable with summary statistics or with only the effect estimates from individual studies.

We also constructed Banksia plots^28^ to visually compare the summary effect estimates and the widths of the 95% CIs. Each original summary effect estimate was treated as the reference value and centred on zero, and its CI was scaled to span a range of one (from −0.5 to 0.5). The corresponding reproduced summary effect estimate and CI were then adjusted by the same amount.^28^ This approach allows easy identification of whether the scaled differences and their intervals are similar or different between the original and reproduced summary estimates. Plots were generated separately for meta-analyses of standardised mean difference, log(RR), log(OR), and log(HR).

#### Comparison of P values

We compared P values from the statistical test of the summary estimate between the original and reproduced meta-analyses by categorising the P values based on commonly used levels of significance (P<0.01; 0.01≤P<0.05; 0.05≤P<0.1; or P≥0.1). We calculated the percentage of meta-analyses where disagreement existed in the categories of significance for the summary estimate.

#### Classification of challenges with reproducing meta-analyses

We used an inductive approach^29^ to identify and categorise the challenges in obtaining data and code, and reproducing the meta-analyses. One author (P-YN) read the notes recorded by all investigators that described the challenges and assigned a category that captured the meaning and content of the text. As each subsequent note was read, existing categories were reviewed and revised, and new categories were added, when necessary. All categories assigned were reviewed by another author (MP) and any discrepancies were resolved by discussion. We present the frequency (percentage) for each category.

### Patient and public involvement

This is a meta-research study that uses secondary data from published journal articles and does not involve recruiting patients. Therefore, patients and/or the public were not involved in the design, or conduct, or reporting, or dissemination plans of this research. Results of this study will be dissemminated to the research community through academic conferences.

## Results

Of the initial sample of 300 reviews, four (1%) were excluded because the reviews were retracted after inclusion in our previous study (n=2) or because a Bayesian network meta-analysis was used (n=2). The remaining 296 reviews met the inclusion criteria and formed the overall sample of the study. Of these, 175/296 (59%) reviews had a forest plot from RevMan and were considered inherently reproducible. The remaining 121/296 (41%) constituted the reproduction sample (online supplemental data S5).

### Characteristics of included reviews

On average, the reviews in the overall sample searched a median of four databases (interquartile range 3-5), included a median of 12 studies (8-21), with the index meta-analyses having a median of six studies (4-11) (table 1). Half of the systematic reviews (n=148/296, 50%) had a corresponding author based in one of three countries: China (32%), US (10%), and UK (8%). Eight reviews (3%) were Cochrane reviews. In 242/296 reviews (82%), the authors stated that the review was reported in accordance with a reporting guideline.

**Table 1:** Characteristics of included reviews in overall sample (n=296)

Characteristics	Frequency (%) or median (IQR)
No of databases searched	4 (3-5)
No of studies included in review	12 (8-21)
No of studies in index meta-analysis of review	6 (4-11)
Country of corresponding author:	
China	96 (32)
USA	29 (10)
UK	23 (8)
Source of funding:	
Non-profit	112 (38)
For-profit	2 (1)
Both non-profit and for-profit	3 (1)
No funding	95 (32)
Not reported	84 (28)
ICD-11 category investigated:	
Endocrine, nutritional, or metabolic diseases	36 (12)
Diseases of the digestive system	34 (11)
Diseases of the musculoskeletal system or connective tissue	34 (11)
Diseases of the circulatory system	30 (10)
Other	162 (55)
Type of intervention:	
Drug	100 (34)
Non-drug	187 (63)
Both	9 (3)
Area of intervention:	
Health	290 (98)
Behavioural	28 (9)
Educational	4 (1)
Social	5 (2)
Cited a report guideline	242 (82)
Cochrane review	8 (3)

ICD-11, international classification of diseases, 11th revision; IQR, interquartile range; UK, United Kingdom; USA, United States of America.

The reviews covered a wide range of topics: health, behavioural, social, and educational interventions, as well as drug treatment and non-drug treatment interventions. Of 24 ICD-11 (international classification of diseases, 11th revision) categories of diseases and conditions, our overall sample of reviews captured 23 categories.

### Correspondence with original systematic reviewers

We invited 118 corresponding authors of 121 reviews (three were authors of more than one review) in the reproduction sample to provide a data file and analytic code for the index meta-analysis of their review. Of these 118 authors, 22 (19%) provided some or all of the requested data (average 37 days, range 1-83, from the date of first invitation to the date of receipt of the data) and five (4%) responded to the invitation but declined to provide data. Delivery of invitation emails to the corresponding author’s email address failed for eight (7%) of 118 reviews, or were successfully delivered with no response from the authors for 83 (70%) of 118 reviews.

### Availability of data and code

Table 2 summarises the availability of data and analysis code. Of the 121 reviews in the reproduction sample, 95% (n=115, 95% CI 90% to 98%) had study level summary statistics or study level effect estimates available in the review report or from communication with the authors. Fewer than half of these reviews had summary statistics available (n=51; 42%, 33% to 51%) whereas more than half had only study level effect estimates available (n=64; 53%, 44% to 62%). Online supplemental data S6 has more information on the statistical methods and meta-analytic results. Of the 87 systematic reviews conducted with software packages where code could have been used (eg, Stata or R), 15 (17%, 10% to 27%) had analytic code available. Of these 15 reviews, analytic code was publicly accessible for two (2%) of 87 reviews, whereas the code was provided by the authors for the remaining 13 (15) of 87 reviews.

**Table 2:** Availability of data and analysis code

Item	Frequency (No/total No (%))	
	Overall sample	Reproduction sample*
Summary statistics:		
Provided in article	199/296 (67)	42/121 (35)
Provided in author's correspondence	9/296 (3)	9/121 (7)
Not provided in article or in author's correspondence	7/296 (2)	7/121 (6)
Not provided in article and no response from author	81/296 (27)	63/121 (52)
Effect estimates:		
Provided in article	288/296 (97)	113/121 (93)
Provided in author's correspondence	1/296 (<0.5)	1/121 (1)
Not provided in article and no response from author	7/296 (2)	7/121 (6)
Analytic code (in 87 reviews where code could have been used):		
Provided in article	2/87 (2)	2/87 (2)
Provided in author's correspondence	13/87 (15)	13/87 (15)
Not provided in article or in author's correspondence	10/87 (11)	10/87 (11)
Not provided in article and no response from author	62/87 (71)	62/87 (71)

* Reproduction sample excluded 175 reviews that had a forest plot generated from Review Manager for the index meta-analysis. In the descriptive analysis of the overall sample, the summary statistics or individual studies' effect estimates of these 175 reviews were considered to be provided in the article.

### Reproducibility of results

For the reproduction sample, we could attempt reanalysis of 92% of the index meta-analyses (n=111/121), with 74% (n=89/121) not requiring input from the original authors and 18% (22/121) involving the authors who provided some or all of the requested data or code (table 3). Ten (8%) of the 121 meta-analyses could not be reproduced because no study level data were available or the available data were not usable (eg, the analytic code could not be run with the dataset). In 15 of the 22 reviews where the authors were involved, materials provided by the author improved the usability of the dataset compared with using materials provided in the article alone. For the other seven reviews, the data or analytic code provided no further information than that provided in the published article.

**Table 3:** Reproducibility of meta-analysis results

Item	Frequency (No/total No (%))	
	Overall sample	Reproduction sample*
Usability of data:		
Not available or not usable for reanalysis	10/296 (3)	10/121 (8)
Usable, reanalysis could be attempted	286/296 (97)	111/121 (92)
Using effect estimates only, summary estimates not provided	80/296 (27)	62/121 (51)
Using effect estimates only, summary estimates provided but not usable	9/296 (3)	9/121 (7)
Using either summary estimates or effect estimates	197/296 (67)	40/121 (33)
With involvement of author†	22/296 (7)	22/121 (18)
Without involvement of author†	264/296 (89)	89/121 (74)
Usefulness of data or code, or both, provided by author (for reviews with author involvement):		
Author provided data and improved usability	4/22 (18)	4/22 (18)
Author provided data and code and both improved usability	2/22 (9)	2/22 (9)
Author provided data and code, only code improved usability	9/22 (41)	9/22 (41)
Author provided data and code, but neither improved usability	1/22 (5)	1/22 (5)
Author provided data but did not improve usability	5/22 (23)	5/22 (23)
Author provided code but did not improve usability	1/22 (5)	1/22 (5)
Index meta-analysis classified as results fully reproducible:	279/296 (94)	104/121 (86)
Without involvement of author†	257/296 (87)	82/121 (68)
With involvement of author†	22/296 (7)	22/121 (18)
Index meta-analysis classified as results not fully reproducible:	7/296 (2)	7/121 (6)
Without involvement of author†	7/296 (2)	7/121 (6)
With involvement of author†	0/296 (0)	0/121 (0)
Index meta-analysis classified as results cannot be reproduced because of missing information	10/296 (3)	10/121 (8)
Index meta-analysis with meaningful differences between original and reproduced results	0/296 (0)	0/121 (0)
Discordance in P value categories between original and reproduced results (in reviews reporting P value of the summary effect)	1/245 (<0.5)	1/65 (2)

* The reproduction sample excluded 175 reviews that had a forest plot generated from Review Manager for their index meta-analyses. In the descriptive analysis of the overall sample, these 175 meta-analyses were considered inherently fully reproducible without involvement of the author; their summary statistics or individual studies' effect estimates were considered usable data for reproduction.

† Involvement of author indicates that the author responded or provided the requested data or analytic code, or both.

In 86% of the index meta-analyses (95% CI 78% to 92%; n=104/121), the results were fully reproducible. In 7/121 meta-analyses (6%, 95% CI 2% to 12%), the results were not fully reproducible (online supplemental data S7). The 10/121 meta-analyses without usable data for reproduction (8%, 95% CI 4% to 15%) were classified as results cannot be reproduced because of missing information. We could fully reproduce 82 meta-analyses (68%, 95% CI 59% to 76%) without involving the original authors. In the sensitivity analysis, with the 5% threshold determining full reproducibility (instead of 10%), 11 meta-analyses previously considered to be fully reproducible were reclassified as not fully reproducible. This brings the total number of not fully reproducible meta-analyses increased from 17/121 (14%) to 28/121 (23%) (online supplemental data S7). We found no meaningful difference between the original and reproduced summary effect estimates that might change the interpretation of the results for any of the 121 meta-analyses.

### Comparison of meta-analysis estimates and confidence intervals

**Figure 1:**
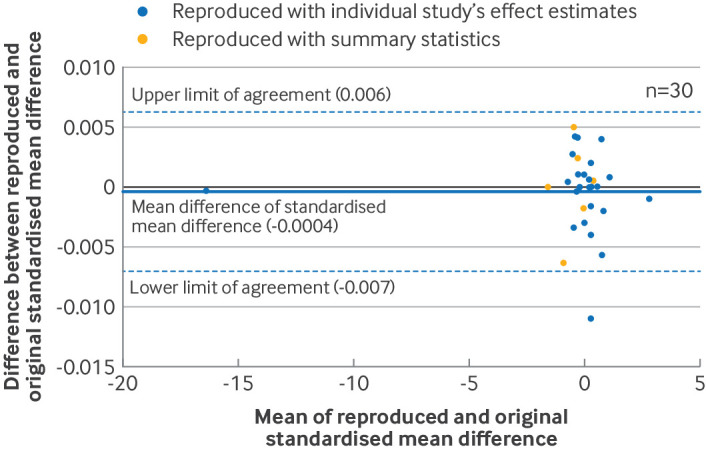
Bland-Altman plot comparing reproduced and original meta-analysis summary effect estimates in the reproduction sample. Differences between original and reproduced standardised mean differences were plotted against the mean of the original and reproduced estimates in 30 meta-analyses of SMD. For Bland-Altman plots that included meta-analyses using Review Manager, see supplementary file S9

**Figure 2:**
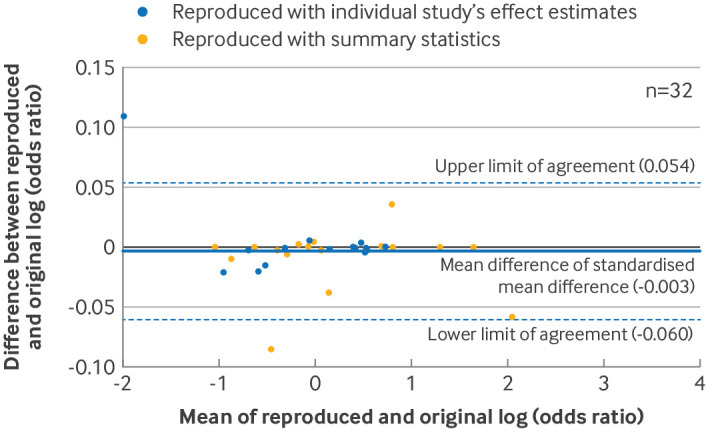
Bland-Altman plot comparing reproduced and original meta-analysis summary effect estimates in the reproduction sample. Differences between original and reproduced log of odds ratios were plotted against the mean of the original and reproduced estimates in 32 meta-analyses of odds ratios. Summary estimates were converted to log scale before analysis. For Bland-Altman plots that included meta-analyses using Review Manager, see supplementary file S9.

**Figure 3:**
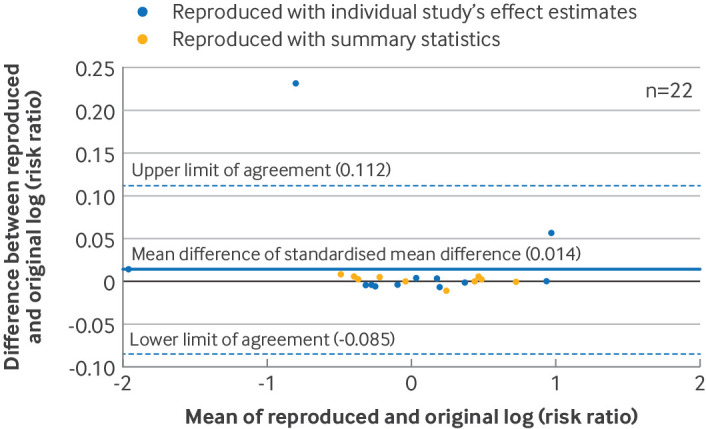
Bland-Altman plot comparing reproduced and original meta-analysis summary effect estimates in the reproduction sample. Differences between original and reproduced log of risk ratios were plotted against the mean of the original and reproduced estimates in 22 meta-analyses of risk ratios. Summary estimates were converted to log scale before analysis. For Bland-Altman plots that included meta-analyses using Review Manager, see supplementary file S9

In the reproduction sample, the average difference in standardised mean difference was −0.0004 (95% limits of agreement −0.007 to 0.006, n=30 index meta-analyses, figure 1), the ratio of odds ratio was 1.00 (95% limits of agreement 0.94 to 1.06, n=32, figure 2), and the ratio of risk ratio was 1.01 (95% limits of agreement 0.92 to 1.12, n=22, figure 3). These results indicated, at most, small differences between the original and reproduced results. An agreement analysis was not undertaken for meta-analyses of hazard ratios because of the small sample size (n=3). Banksia plots also showed no major differences between the reproduced and original meta-analysis summary estimates and their confidence intervals (online supplemental data S8).

### Comparison of P values

In the reproduction sample, 65 (54%) of 121 index meta-analyses reported a P value for the summary effect. Only one meta-analysis (2%, 95% CI 0% to 8%) had a discordant P value on reanalysis (ie, the reproduced P value was > 0.1 whereas the original P value was between 0.05 and 0.1, table 3).

### Challenges during reproduction

As well as the low response rate from the authors of the original reviews, we encountered several other challenges during our reproduction attempts (table 4). These challenges were: insufficient or unclear information provided in some of the original reviews, especially for how particular effect estimates (eg, rate ratios and incidence rate ratios) were computed; data unavailable (ie, absence of study level effect estimates) or absence of summary statistics for a meta-analysis method that required summary statistics (eg, Mantel-Haenszel method); low quality forest plots (eg, low resolution, only presenting values in figure format, or typographical errors); and discrepancies in the reported statistical methods or results (eg, discrepancies between results presented in text *v* results from the forest plot).

**Table 4:** Challenges encountered during reproduction of meta-analyses

Theme	Challenge	No of meta-analyses affected*
Insufficient or unclear instructions on statistical methods	Insufficient information was provided on how the effect estimate for individual studies was calculated.	4
	The meta-analysis method was not clearly reported.	3
	Adjusted odds ratios were reported without the underlying data or an explanation of the adjustment.	3
	Only a subset of the studies were included in the meta-analysis, without instruction on which studies should be excluded and why.	2
	Insufficient information was provided on transformations of data before meta-analysis.	2
Data unavailable	Both summary statistics and effect estimates were unavailable to reproduce the meta-analysis.	6
	Summary statistics were required to reproduce the meta-analysis with a particular method but were unavailable.	4
	Analytic code was provided without the suitable dataset to run.	3
	Correlation coefficients between before-after measurements were not available for meta-analysis of before-after standardised mean differences.	2
	Effect estimates for some studies were generated with individual patient data, which were not available.	1
Low quality forest plots	Low quality forest plot (eg, low resolution, values of effect estimates or 95% CI not presented) hindered data extraction.	3
	Typographical error on the forest plot prevented accurate comparison between original and reproduced results.	1
Discrepancies in the reported statistical methods or results	Discrepancies observed between results presented in different parts of the article (eg, forest plot *v* results in the text).	2
	Reproduction suggested standardised mean difference was used in meta-analysis but the report specified that mean difference was used.	1

* Some meta-analyses were affected by more than one challenge.

CI, confidence interval.

## Discussion

### Principal findings

We found that reproduced meta-analytic results were almost always the same as the original results, and any discrepancies observed were unlikely to change the conclusion of the review. For example, for meta-analyses of standardised mean difference, the average discrepancy (−0.0004) and the limits of agreement (where 95% of discrepancies would be expected to lie) were much lower than the conventional threshold for a small effect (0.2).^30^ Our rate for meta-analyses not fully reproducible at the 5% threshold (23%) was similar to another replication study that used the same threshold (18%).^14^

Our findings illustrate some gaps in reporting that the Preferred Reporting Items for Systematic Reviews and Meta-Analyses (PRISMA) 2020 guideline^31^ tried to answer. Only 35% of the reviews in the reproduction sample had study level summary statistics available in the article. A similar frequency (37%) was observed in another study where the authors attempted to reproduce meta-analyses of diagnostic test accuracy data but often did not have the data necessary to reproduce the 2×2 analysis for each study.^22^ Therefore, for most of our reproduction attempts, we relied on study level effect estimates and measures of precision rather than summary statistics. This finding highlights the need for systematic reviewers to always provide summary statistics for each included study where possible, as recommended in the PRISMA guideline,^31^ preferably in extractable formats such as an Excel file.

Systematic reviewers should provide details of the meta-analysis models and methods used to ensure reproducibility. A non-specific description, such as stating that “we used random effects meta-analysis,” without specifying the software or package used, may hinder readers from inferring the correct methods because default settings can vary across software programmes (online supplemental data S4). We also encourage review authors to include details of the model and statistical method within the code itself. For example, when writing code for a random effects meta-analysis with the metan command in Stata, specifying model(dlaird) instead of model(random) makes it clear for readers that the DerSimonian and Laird estimator for between study variance was used. Finally, we recommend that software developers enhance transparency by ensuring that selected meta-analysis options are displayed by default on forest plots, allowing users to verify the methodological choices made during analysis.

Although authors often state that data will be provided on request,^32^ our attempts to obtain data from authors were often unsuccessful, with only 19% of authors responding, consistent with previous rates.^13^ We encourage systematic reviewers to routinely make the data files underlying the results of the meta-analysis publicly available on open access platforms (eg,Open Science Framework, Zenodo, GitHub, or medRxiv). Doing so will reduce the time required for data extraction and allow faster updates of systematic reviews and clinical guidelines. Box 1 summarises our recommendations to improve reproducibility of meta-analyses.

Box 1 **Recommendation to improve reproducibility of meta-analyses**

1. Store the datasets or analytic code, or both, in a publicly available, FAIR (findable, accessible, interoperable, and reusable) aligned repository, and provide links in the data availability statement of the results report.
2. Provide the summary statistics used to generate study effect estimates (where applicable) on the forest plot or in a separate table.
3. If individual effect estimates (rather than summary statistics) were entered in the meta-analysis:
  - clarify whether the effect estimates were extracted directly from the included studies or calculated separately by the reviewers and
  - provide a brief description of how the effect estimates were calculated, for example,
    - for standardised mean differences, clarify the method used to estimate standardised mean difference (Cohen's d or Hedge's g);
    - for binary data, clarify the continuity correction method for zero cells;
    - for hazard ratios, clarify whether ratios and their variance were indirectly estimated from Kaplan-Meier curves or other data reported in the text; and
    - for before and after measurements, provide correlation coefficients.
4. Report the software and command or package used for meta-analysis and the version numbers of both the software and the respective command or package.
5. When writing the analytic code, spell out all statistical options even if they are the default options of the software.
6. Ensure the publicly shared dataset is compatible with the analytic code.

### Strengths and limitations of this study

We used random sampling to select the systematic reviews, which makes our findings more generalisable. We examined the reproducibility of meta-analyses by reanalysing the meta-analytic data, rather than by assessing the completeness of reporting. Although similar projects attempted to reproduce meta-analyses of standardised mean differences,^19 21 33^ we also reproduced meta-analyses of mean differences, odds ratios, risk ratios, and hazard ratios.

Our study had some limitations. For systematic reviews where information on the software used was unclear, we used R's meta as the default software and package for reproduction. This decision was based on our assumption of the most popular and accessible software, which could be different from that used in the original review. Also, we did not implement version control for packages in R because none of the reviews indicated the version of the R packages used.

### Future research

Our study did not include bayesian meta-analyses and meta-analyses of individual participant data, which have different requirements for type of data, statistical techniques, and software used. Future reproduction studies might examine these types of meta-analyses. For an individual participant data meta-analysis, exploring the challenges in obtaining access to the underlying individual participant data in the context of reproduction would be useful.^34^

### Conclusions

The findings of our study suggested that the results of meta-analyses could be reliably replicated if the original data or analytic code, or both, could be obtained, or if the necessary data were accessible in the review. The response rate from the original systematic reviewers for sharing of data or analytic code, or both, was low however, and we often had to extract the necessary data from the review reports. Making data files and analytic code publicly available will facilitate future investigations of reproducibility of meta-analyses and allow updates of reviews to be completed more quickly. We have provided recommendations to systematic reviewers and other stakeholders to make this process more efficient.

## Supplementary material

10.1136/bmjmed-2025-002024 online supplemental file 1

## Data Availability

Data are available in a public, open access repository.
