# Supplementary material for: Reproducibility of meta-analytic results in systematic reviews of interventions: meta-research study
Source: BMJ Med. 2025 Nov 30;4(1):e002024. doi: 10.1136/bmjmed-2025-002024 (PMC12684188; doi:10.1136/bmjmed-2025-002024)
Supplement: Supplementary file 2 [file bmjmed-2025-002024.reviewer_comments.pdf]

PEER REVIEW HISTORY

BMJ Medicine publishes all reviews undertaken for accepted manuscripts. Reviewers are asked to complete a checklist review form and are provided with free text boxes to elaborate on their assessment. These free text comments are reproduced below.

This paper was submitted to another journal from BMJ but declined for publication following peer review. The authors addressed the reviewers' comments and submitted the revised paper to BMJ Medicine. The paper was subsequently accepted for publication at BMJ Medicine.

ARTICLE DETAILS

**TITLE (PROVISIONAL)**	Reproducibility of meta-analytic results in systematic reviews of interventions: a meta-research study
**AUTHORS**	Nguyen, Phi-Yen; McKenzie, Joanne; Alqaidoom, Zainab; Hamilton, Daniel; Moher, David; Page, Matthew

VERSION 1 - REVIEW

**REVIEWER NAME**	Dewi, Ayu Putu Madri
**REVIEWER AFFILIATION**	Amsterdam UMC
**REVIEWER CONFLICT OF INTEREST**	None
**DATE REVIEW RETURNED**	23-May-2025

**GENERAL COMMENTS**	Manuscript Title: Reproducibility of meta-analytic results in systematic reviews of interventions: a meta-research study<br><br>Dear authors,<br>I am an Open Science researcher, who has checked your manuscript for its adherence to a number of Open Science practices.<br>Below you find the checklist that I used to assess your manuscript for adherence to a number of these practices.<br><br>Based on this assessment, I would suggest the following refinements/additions:<br>1. Availability of a protocol: OK<br>2. (Pre-)registration: OK<br>3. Open Materials: OK<br>5. Open Data: OK<br>6. Open Code: OK<br>6. Citations: OK<br>7. Software and tools used are described: OK<br>8. Open access publication: OK<br>9. Reporting checklist provided: Please provide the correct completed reporting checklist (see e.g. https://www.equator-network.org/reporting-guidelines/).<br>10. Preprint: Please state explicitly whether and where the preprint of this study can be found.

**REVIEWER NAME**	Siemens, Waldemar
**REVIEWER AFFILIATION**	Institute for Evidence in Medicine, Medical Center - University of Freiburg / Medical Faculty - University of Freiburg, Germany

**REVIEWER CONFLICT OF INTEREST**	None
**DATE REVIEW RETURNED**	23-May-2025

**GENERAL COMMENTS**	Summary<br>Thank you for having the opportunity to review the manuscript "Reproducibility of meta-analytic results in systematic reviews of interventions: a metaresearch". The topic reproducibility of meta-analytic results is clearly relevant across different medical and non-medical fields.<br>The introduction is clear and well-contextualized, and the methods are well described, with appropriate justifications for deviations from the protocol.<br>However, certain aspects would benefit from further clarification. Some points were not very clear and could be explained in more detail, e.g., it was not clear if multiple meta-analyses per review were analysed. Additionally, some methodological decisions such as the choice for 10% difference as reproducibility threshold and the role of MIDs in interpretation could be elaborated upon in the discussion.<br>The recommendations to improve reproducibility of meta-analyses added in the discussion are very helpful and the overall conclusions are clear and appropriate.<br>I encourage the authors to revise accordingly and would be happy to reassess the manuscript upon resubmission.<br><br><br>Major comments<br><br>#1<br>Were multiple meta-analyses per systematic review reproduced? If yes, how did you address the correlation within the meta-analyses of a systematic review? (intra-review correlation)<br>Or was it one meta-analysis per systematic review? If yes, how did you chose the meta-analysis if more than one meta-analysis was available in a systematic review?<br><br>#2<br>„fully reproducible if there was less than a 10% difference" – Is there a rationale why the authors chose 10%? Have you thought about a sensitivity analysis with e.g. 5%? Do you think the results will remain robust?<br><br>#3<br>A meaningful effect was defined by opposite direction and inclusion of the null effect. Depending on the outcome crossing (available and validated) minimal clinically important differences could also be considered as meaningful criterion, do you agree? The differences of original and reproduced effects were small but do you think individual MIDs could change the interpretation of your result? Obviously, such an analysis would be very complex because you would have to think of a MID for each outcome.<br><br>#4<br>What about the (clinical) characteristics of the included systematic reviews, e.g. patients? It would be helpful to show them to give context, and to strengthen or attenuate the claim on generalizability made in the discussion.

	Minor comments<br><br>Abstract:<br>#1<br>“sufficiently similar results when reanalysing the same data using the same methods” – why sufficiently similar? With same data and the same methods, I would assume similar results. Differences would then probably only occur due to different rounding of decimal places.<br><br>#2<br>“its 95% confidence interval (CI)” – You may add that you refer to the width of the CI to be more precise (as you specified it in the methods).<br><br><br>Introduction:<br>#3<br>“In some cases, the summary statistics used to calculate individual study effect estimates are not reported” – What is meant by “summary statistics”? Events, total, mean, standard deviation? Please give an example.<br><br>#4<br>“These scenarios mean that assumptions might need to be made about the data and meta-analysis methods in reproduction attempts.” – Could you give an example for an assumption?<br><br><br>Methods:<br>#5<br>“until the target sample size of 300 systematic reviews was reached” – What is the rationale for choosing 300 systematic reviews?<br><br>#6<br>“Data extraction was carried out by one investigator (P-YN), using a standardised data extraction<br>form in Microsoft Excel.” – This seems to be a limitation. However, error in data extraction would reduce reproducibility resulting rather in an underestimation of the reproducibility. In other words, it strengthens the result that reproducibility was high although data extraction was carried out only by one investigator.<br><br>#7<br>“above versus below the null value” – would it be probably more precise writing “null effect” and add that this is 1 for ratio measures and 0 for difference measures?<br><br>#8<br>“We present the frequency (with percentage) of each code.” – Consider replacing “code” with “category”. From my knowledge “code” in qualitative research refers more to segments of the data and “category” is a higher level grouping of different codes representing the same category.<br><br>#9<br>Were the studies included in the systematic reviews RCTs or also NRSIs? ORs (e.g. from NRSIs) are for example often based on logistic regression so the OR cannot be reproduced by the raw

	summary statistics (event, total). Does this impact on the interpretation of the results?<br><br>Results:<br>#10<br>First paragraph: I think a flow chart would be helpful to visualize the dataset.<br><br>#11<br>"successfully delivered with no response from authors for 83/118 reviews" – Did you contact other authors of a review if the corresponding author did not reply?<br><br>Discussion:<br>#12<br>"and lower than that reported in a previous reproducibility study in psychology" – Could you add the numeric results to enable a quantitative comparison?<br><br>#13<br>"routinely make their data file(s) underlying meta-analysis results publicly available." – You may suggest platforms. This could be helpful for some readers not familiar with platforms like OSF, Zenodo or others.

**REVIEWER NAME**	Reviewer 3
**REVIEWER AFFILIATION**	
**REVIEWER CONFLICT OF INTEREST**	In the past 36 months, I have received funding from NIAAA/NIA, FDA, Arnold Ventures, and Johnson & Johnson (through the Yale Open Data Access Project).
**DATE REVIEW RETURNED**	23-May-2025

**GENERAL COMMENTS**	Thank you for the opportunity to review this manuscript by Nguyen et al., which investigates the reproducibility of meta-analytic results in systematic reviews of interventions. The authors used a sample of 296 systematic reviews of health, social, behavioral, or educational interventions to assess how often meta-analyses can be reproduced. They attempted to replicate each meta-analysis using the same data, computational steps, and analytic code employed in the original review. Among the 296 meta-analyses, 94% were considered fully reproducible (defined as <10% difference between the original and reproduced summary estimate and its 95% CI), 2% were not fully reproducible, and 3% could not be assessed due to insufficient data. The authors conclude that meta-analytic results are largely consistent upon reproduction, though response rates to requests for data and code were low.<br><br>The authors should be commended for adhering to best practices in research transparency, including the pre-registration of their study and the clear reporting of methods. Systematic reviews and meta-analyses can significantly influence clinical and policy decisions, making reproducibility a critical aspect of their validity and utility.<br><br>However, there are several overarching gaps in this evaluation that limit its strength, generalizability, and clarity. I have summarized the primary thematic gaps below:

1. The manuscript focuses on methods reproducibility—whether the same procedures applied to the same data yield consistent results. It does not assess results reproducibility, which would involve independent teams using new data to replicate findings. While this focus is not necessarily a limitation, the manuscript should clearly distinguish between these concepts (e.g., Goodman et al., Sci Transl Med, 2016) to avoid overstating generalizability. Given the large number of reviews with RevMan analyses, which were automatically classified as being fully reproducible, one could argue that an evaluation of results reproducibility would have been more informative. When it comes to methods reproducibility, it's almost an evaluation focused on transparency – how many articles report summary effect estimates, how many provide data, etc. With nearly 60% of the meta-analyses using RevMan, it almost seems like the focus of this manuscript should be on transparency and not on reproducibility. However, I believe some of this was already covered in the BMJ publication from the same authors in 2022.

2. The study includes a heterogeneous and slightly dated (2020) sample of reviews across multiple domains. The study uses a previously assembled sample of systematic reviews addressing health, social, behavioral, and educational interventions. While this breadth reflects the authors' intent to assess a wide range of meta-analyses, the heterogeneity in intervention type and review quality could make it harder to draw generalizable conclusions. In the sample, what are the types of interventions and outcomes? How many were Cochrane reviews? It would seem like this evaluation would be more informative if the authors focused on meta-analyses that were used to inform guidelines.

3. The meta-analyses conducted using RevMan were excluded from reproducibility attempts under the rationale that the same software applied to the same data would yield identical results (page 7 line 36: "We attempt to reproduce the index meta-analysis in all systematic reviews included in our sample that used software other than RevMan"). However, the reporting related to the RevMan and non-RevMan samples is rather confusing, as the denominator jumps around in the Results. The manuscript would benefit from clearer reporting around denominators. There are also a few important questions related to this decision: what proportion of all meta-analyses in the peer-reviewed literature are Cochrane meta-analyses (which likely have RevMan) or non-Cochrane meta-analyses that use RevMan? The proportion classified as reproducible due to using RevMan may be inflated if this sample includes a larger proportion of Cochrane meta-analyses than the proportion of these across the entire health intervention literature. It is worth noting that 60% of the sample presented a forest plot generated by RevMan and were considered inherently reproducible. This demonstrates the limitation of focusing on methods reproducibility. If these evaluations are considered fully reproducible, the more interesting question is whether an individual author could identify the same sample studies and results using different methods (results reproducibility).

4. The concluding statements are broad. Specifically, the claim that "most meta-analyses could be reproduced using available data" could be more cautiously phrased, given that only non-RevMan reviews were truly reproduced and code availability was not determined for these. It may be more accurate to state that among

	reviews where data and code were accessible, the majority of meta-analyses yielded consistent results when re-analyzing the data using the provided code or methods.

VERSION 1 – AUTHOR RESPONSE

REVIEWER 2

R2Q1. Were multiple meta-analyses per systematic review reproduced? If yes, how did you address the correlation within the meta-analyses of a systematic review? (intra-review correlation). Or was it one meta-analysis per systematic review? If yes, how did you chose the meta-analysis if more than one meta-analysis was available in a systematic review?

R: We only reproduced one meta-analysis per systematic review. We noted this in the Methods section: "In each review in this sample, we attempted to reproduce the first meta-analysis that was mentioned in the abstract or the Results section, hereafter called the 'index meta-analysis'."

R2Q2. "fully reproducible if there was less than a 10% difference" – Is there a rationale why the authors chose 10%? Have you thought about a sensitivity analysis with e.g. 5%? Do you think the results will remain robust?

R: We have now conducted a sensitivity analysis with a 5% threshold and presented the results: "In the sensitivity analysis, when using the 5% threshold to determine full reproducibility (instead of 10%), nine meta-analyses previously considered fully reproducible were re-classified as partially reproducible. This brings the total number of not fully-reproducible meta-analyses from 17/121 (14%) to 28/121 (23%) (Supplementary File S7)."

R2Q3. A meaningful effect was defined by opposite direction and inclusion of the null effect. Depending on the outcome crossing (available and validated) minimal clinically important differences could also be considered as meaningful criterion, do you agree? The differences of original and reproduced effects were small but do you think individual MIDs could change the interpretation of your result? Obviously, such an analysis would be very complex because you would have to think of a MID for each outcome.

R: As the reviewer correctly pointed out, there are several challenges with using MIDs in this study: (1) for many outcomes, a validated and widely accepted MID does not exist; (2) identifying a MID for each outcome would require clinical expertise and extensive research for each of the 121 meta-analyses, which is very resource intensive.

Moreover, given that the discrepancies we observed were very small, it is unlikely that additional considerations for MID would change our overall conclusion about the reproducibility of meta-analyses in our sample.

R2Q4. What about the (clinical) characteristics of the included systematic reviews, e.g. patients? It would be helpful to show them to give context, and to strengthen or attenuate the claim on generalizability made in the discussion.

R: We have added a new Table 1 which provides characteristics of the overall sample of systematic reviews.

R2Q5. "sufficiently similar results when reanalysing the same data using the same methods" – why sufficiently similar? With same data and the same methods, I would assume similar results. Differences would then probably only occur due to different rounding of decimal places.

R: Yes, "sufficiently similar" allows for potential small discrepancies due to rounding of decimal places.

R2Q6. "its 95% confidence interval (CI)" – You may add that you refer to the width of the CI to be more precise (as you specified it in the methods).

R: We have changed it to "95% confidence interval (CI) *widths*".

R2Q7. "In some cases, the summary statistics used to calculate individual study effect estimates are not reported" – What is meant by "summary statistics"? Events, total, mean, standard deviation? Please give an example.

R: We elaborated on summary statistics, as follows: "individual study summary statistics *(i.e. for continuous data, means, standard deviations and sample sizes; and for binary outcomes, number of events and sample sizes)*"

R2Q8. "These scenarios mean that assumptions might need to be made about the data and meta-analysis methods in reproduction attempts." – Could you give an example for an assumption?

R: We provided an example in the following sentence in the Introduction. "For example, if an author reports they used random-effects meta-analysis without further details, there are a myriad of between-study variance estimators and confidence interval methods for the summary effect that could have been used (11,12)." We did not elaborate to keep the Introduction concise, but more specific examples were provided later in the Methods section, (for example, "the meta-analysis method (e.g. inverse variance or Mantel-Haenszel method), the heterogeneity variance estimator used, if applicable (e.g. DerSimonian-Laird or Restricted Maximum Likelihood estimator)").

R2Q9. "until the target sample size of 300 systematic reviews was reached" – What is the rationale for choosing 300 systematic reviews?

R: As noted in the REPRISE protocol (Supplementary File S1, file attached during submission) "including 300 systematic reviews allowed us to estimate the percentage of reviews reporting a particular practice (e.g. reporting the full line-by-line search strategy) to within a maximum margin of error of 6% assuming a prevalence of 50%; for a prevalence of less (or greater) than 50%, the margin of error will be smaller."

R2Q10. "Data extraction was carried out by one investigator (P-YN), using a standardised data extraction form in Microsoft Excel." – This seems to be a limitation. However, error in data extraction would reduce reproducibility resulting rather in an underestimation of the reproducibility. In other words, it strengthens the result that reproducibility was high although data extraction was carried out only by one investigator.

R: We agree.

R2Q11. "above versus below the null value" – would it be probably more precise writing "null effect" and add that this is 1 for ratio measures and 0 for difference measures?

R: We have added this point, as follows: "the null effect – 1 for ratio measures and 0 for difference measures)"

R2Q12. "We present the frequency (with percentage) of each code." – Consider replacing "code" with "category". From my knowledge "code" in qualitative research refers more to segments of the data and "category" is a higher level grouping of different codes representing the same category.

R: We changed "code" to "category".

R2Q13. Were the studies included in the systematic reviews RCTs or also NRSIs? ORs (e.g. from NRSIs) are for example often based on logistic regression so the OR cannot be reproduced by the raw summary statistics (event, total). Does this impact on the interpretation of the results?

R: Some reviews included NRSIs. When effect estimates were not obtained using raw summary statistics (e.g. odds ratios from logistic regression or hazard ratios from Cox Proportional Hazards Model), we reproduced the meta-analyses using the individual study's effect estimates only. This was specified in the Methods section: "If summary statistics were available for each study included in the meta-analysis, we attempted to reproduce the meta-analysis using these values. If only effect estimates and measures of precision were available, we attempted to reproduce the meta-analysis using these values, using the inverse variance method". We attempted to retrieve the analytic code authors used to arrive at these effect estimates, but were unsuccessful in those attempts. Overall, such meta-analysis would still be considered reproducible, with the caveat that we were unable to detect any discrepancy arising from the process of producing these individual study's effect estimates.

In our Results, we distinguished between reproduction using summary statistics and reproduction using individual effect estimates (see Figure 1 – the use of different colour coding), to allow readers to view the results of both types of reproduction.

R2Q14. First paragraph: I think a flow chart would be helpful to visualize the dataset.

R: We added a flow chart (Supplementary File S5).

R2Q15. "successfully delivered with no response from authors for 83/118 reviews" – Did you contact other authors of a review if the corresponding author did not reply?
R: We only contacted authors designated as the corresponding authors. If there were more than one corresponding authors, we contacted all of them.

R2Q16. "and lower than that reported in a previous reproducibility study in psychology" – Could you add the numeric results to enable a quantitative comparison?
R: We omitted this study from discussion as their reproduction process was not equivalent to ours and did not allow a direct quantitative comparison. Instead, we cited another study with more comparable statistics, "Our rate of meta-analyses not fully reproducible at the 5% threshold (23%) is similar to another replication study that used the same threshold (18% to 28%) (14)."

R2Q17. "routinely make their data file(s) underlying meta-analysis results publicly available." – You may suggest platforms. This could be helpful for some readers not familiar with platforms like OSF, Zenodo or others.

R: We added examples, as follows: "We encourage systematic reviewers to routinely make their data file(s) underlying meta-analysis results publicly available, *using open-access platforms (e.g. Open Science Framework, Zenodo or medRxiv).*"

REVIEWER 3

R2Q1. The manuscript focuses on methods reproducibility—whether the same procedures applied to the same data yield consistent results. It does not assess results reproducibility, which would involve independent teams using new data to replicate findings. While this focus is not necessarily a limitation, the manuscript should clearly distinguish between these concepts (e.g., Goodman et al., Sci Transl Med, 2016) to avoid overstating generalizability.

R: We discussed the definition in the Introduction, "Although the definition of reproducibility varies (3), the general consensus is that achieving reproducibility refers to obtaining the same, or reasonably similar, results when reanalysing data collected for a prior study, using the same computational steps and analytic code as the original study (4,5)." and again in the abstract, "The results of meta-analyses should be reproducible; that is, others should obtain sufficiently similar results when reanalysing the same data using the same methods."

R2Q2. Given the large number of reviews with RevMan analyses, which were automatically classified as being fully reproducible, one could argue that an evaluation of results reproducibility would have been more informative. When it comes to methods reproducibility, it's almost an evaluation focused on transparency – how many articles report summary effect estimates, how many provide data, etc. With nearly 60% of the meta-analyses using RevMan, it almost seems like the focus of this manuscript should be on transparency and not on reproducibility. However, I believe some of this was already covered in the BMJ publication from the same authors in 2022.

R: We have rewritten the Results section to put more focus on the reviews not conducted using Review Manager (see our response to R1Q2).

The focus on this manuscript is still on our reproducibility efforts. Information on availability of data only serves to highlight the challenges we encountered during reproduction and provide context to our findings.

Transparency in reporting has previously been addressed in the first study of REPRISE project and is not covered in the current paper (Nguyen PY, Kanukula R, McKenzie JE et al. Changing patterns in reporting and sharing of review data in systematic reviews with meta-analysis of the effects of interventions: cross sectional meta-research study. bmj. 2022 Nov 22;379).

R2Q3. The study includes a heterogeneous and slightly dated (2020) sample of reviews across multiple domains. The study uses a previously assembled sample of systematic reviews addressing health, social, behavioral, and educational interventions. While this breadth reflects the authors' intent to assess a wide range of meta-analyses, the heterogeneity in intervention type and review quality could make it harder to draw generalizable conclusions.

In the sample, what are the types of interventions and outcomes? How many were Cochrane reviews? It would seem like this evaluation would be more informative if the authors focused on meta-analyses that were used to inform guidelines.

R: The heterogeneity in intervention types and review quality is not a limitation but rather a reflection of the real-world landscape of systematic reviews. Our diverse sample is representative of this varied landscape. Therefore, we consider the findings more, not less, generalisable. More importantly, the generalisability of the findings is supported by the fact that we systematically searched multiple bibliographic databases and used random sampling to select the included reviews.

With regards to the second point, we have added Table 1 ("Characteristics of included reviews in the overall sample (N=296)". While not all included reviews were formally cited in guidelines, it is standard practice for policymakers to conduct a systematic literature search as part of decision-making, and any of these systematic reviews could potentially contribute to the evidence base of their respective field.

R2Q4. The meta-analyses conducted using RevMan were excluded from reproducibility attempts under the rationale that the same software applied to the same data would yield identical results (page 7 line 36: "We attempt to reproduce the index meta-analysis in all systematic reviews included in our sample that used software other than RevMan"). However, the reporting related to the RevMan and non-RevMan samples is rather confusing, as the denominator jumps around in the Results. The manuscript would benefit from clearer reporting around denominators.

R: We have rewritten the paper to focus our results on the sample of 121 reviews, to avoid confusion. Please refer to our response to R1Q2.

R2Q5. There are also a few important questions related to this decision: what proportion of all meta-analyses in the peer-reviewed literature are Cochrane meta-analyses (which likely have RevMan) or non-Cochrane meta-analyses that use RevMan? The proportion classified as reproducible due to using

RevMan may be inflated if this sample includes a larger proportion of Cochrane meta-analyses than the proportion of these across the entire health intervention literature.

R: We added this information in Table 1 and the text: “Eight reviews (3%) were Cochrane reviews.” As such, the proportion of Cochrane meta-analyses is unlikely to inflate the reproducibility of our sample.

R2Q6. It is worth noting that 60% of the sample presented a forest plot generated by RevMan and were considered inherently reproducible. This demonstrates the limitation of focusing on methods reproducibility. If these evaluations are considered fully reproducible, the more interesting question is whether an individual author could identify the same sample studies and results using different methods (results reproducibility).

R: The primary goal of this particular study was to isolate issues in redoing the analysis itself and determine if it is a significant barrier to reproducibility. Please refer to our response to R1Q4 for the full response.

R2Q7. The concluding statements are broad. Specifically, the claim that “most meta-analyses could be reproduced using available data” could be more cautiously phrased, given that only non-RevMan reviews were truly reproduced and code availability was not determined for these. It may be more accurate to state that among reviews where data and code were accessible, the majority of meta-analyses yielded consistent results when re-analyzing the data using the provided code or methods.

R: We have rewritten the conclusion, as follows: “Our study suggests that meta-analysis results could be reliably replicated if the original data and/or analytic code could be obtained or if the necessary data was accessible in the review.”
